# A New Approach for Reducing Virtual Reality Sickness in Real Time: Design and Validation Study

**DOI:** 10.2196/36397

**Published:** 2022-09-27

**Authors:** JuHye Won, Yoon Sang Kim

**Affiliations:** 1 BioComputing Lab Department of Computer Science and Engineering Korea University of Technology and Education Cheonan-si Republic of Korea; 2 BioComputing Lab, Institute for Bio-engineering Application Technology Department of Computer Science and Engineering Korea University of Technology and Education Cheonan-si Republic of Korea

**Keywords:** virtual reality, VR, VR sickness, VR sickness reduction method, simulator sickness questionnaire, SSQ, visual guide, field of view, serious game, VR sickness reduction, VR content, technology, digital health

## Abstract

**Background:**

Recently, technology that provides virtual reality (VR) content based on streaming services has been rapidly developed. However, there have been few studies to reduce VR sickness that occurs while the user watches VR content while wearing a head-mounted display (HMD) in real time.

**Objective:**

Based on this background, we propose a new approach to measure and reduce VR sickness that occurs while the user watches VR content while wearing an HMD in real time.

**Methods:**

The proposed approach is to apply VR sickness reduction methods in accordance with the user’s real-time VR sickness level. Three methods that are known to be effective in reducing VR sickness and a single type of VR content were used to examine the effectiveness of the proposed approach, which was confirmed by the experimental results.

**Results:**

Our results show that VR sickness significantly decreased when a new approach was applied to VR content (in all cases, *P*<.05).

**Conclusions:**

From our results, it was confirmed that VR sickness could be measured without wearing additional equipment, and its reduction method could be applied in real time in accordance with the user’s condition by the proposed approach in this paper.

## Introduction

Recently, technology that provides virtual reality (VR) content based on streaming services (such as YouTube VR, Netflix, etc) have been rapidly developed. Users of head-mounted displays (HMDs) are also increasing, and according to ResearchAndMarkets.com [1], the global HMD market is expected to grow by more than US $36 billion by 2026. However, VR sickness that occurs while the user watches VR content wearing an HMD does not have a positive effect on the proliferation of VR content. To solve this problem, research in the following 3 directions is being conducted.

The first direction is the identification of the cause of VR sickness. Several studies confirmed that speed [2], watching time [3], pitch, and roll rotation [4] affect VR sickness. Another study confirmed that the level of VR sickness varies depending on gender [5]. In addition, studies [6-8] were conducted to confirm the correlation between VR sickness and VR content’s elements, devices, and human factors.

The second direction is the derivation of the VR sickness measurement method. VR sickness measurement methods are divided into subjective and objective methods. Subjective methods were conducted through a survey such as simulator sickness questionnaires (SSQs) [9-11], the Motion Sickness Susceptibility Questionnaire (MSSQ) [12], Game Experience Questionnaire [13,14], Immersive Tendencies Questionnaire, and Presence Questionnaire [14]. Furthermore, in the objective method, studies were conducted to predict VR sickness using biological signals such as an electrocardiograms, electrodermal activity, electrooculogram, and breathing [9,10,15]. However, it is difficult to apply a VR sickness measurement method using biological signals to general users because additional equipment should be used. Furthermore, subjective measurement methods require surveys, and it is difficult to measure VR sickness in real time while VR content is being played.

The third direction is the derivation of VR sickness reduction methods. According to Singla et al [16] who conducted VR sickness reduction studies in terms of hardware, it was confirmed that HTC Vive provides an environment with a lower level of VR sickness than Oculus Rift. However, it is difficult to conclude that specific hardware is more effective in reducing VR sickness because new HMDs are constantly being developed. Therefore, studies are being conducted to reduce VR sickness in terms of content. A study was conducted to reduce VR sickness using the visual effects of VR content [17], and other studies have attempted to reduce VR sickness by applying a virtual human nose as an earth-fixed grid to the VR content [18,19]. A virtual human nose technique features the tip of a nose being fixed at the center-bottom of a VR user’s view, acting as a rest-frame, which the brain can use to make natural spatial adjustments, thus reducing simulator sickness [18]. In addition, “Virtual Guiding Avatar” [20] combines various motion attributes with an independent visual background and Dynamic field of view (FOV) modification technology [21], which partially limits the user’s FOV developed to reduce VR sickness. However, the above methods are only applied in advance in the content development step but do not apply VR sickness reduction methods in real time to suit the user’s condition while VR content is being played.

As such, few studies have attempted to reduce VR sickness that occurs while the user watches VR content wearing an HMD in real time. Therefore, a new approach is proposed to measure and reduce VR sickness that occurs while the user watches VR content while wearing an HMD in real time. The proposed approach uses the VR sickness response, which is the result of the direct response of VR sickness that occurs while the user watches VR content wearing an HMD. Three methods that are known to be effective in reducing VR sickness have been used to examine the effectiveness of the proposed approach, which is confirmed by the experimental results. Furthermore, based on our results, the effect of the new approach for real-time VR sickness reduction and the effect of the VR sickness response are discussed.

## Methods

### A New Approach for VR Sickness Measurement and Reduction

**Figure 1:**
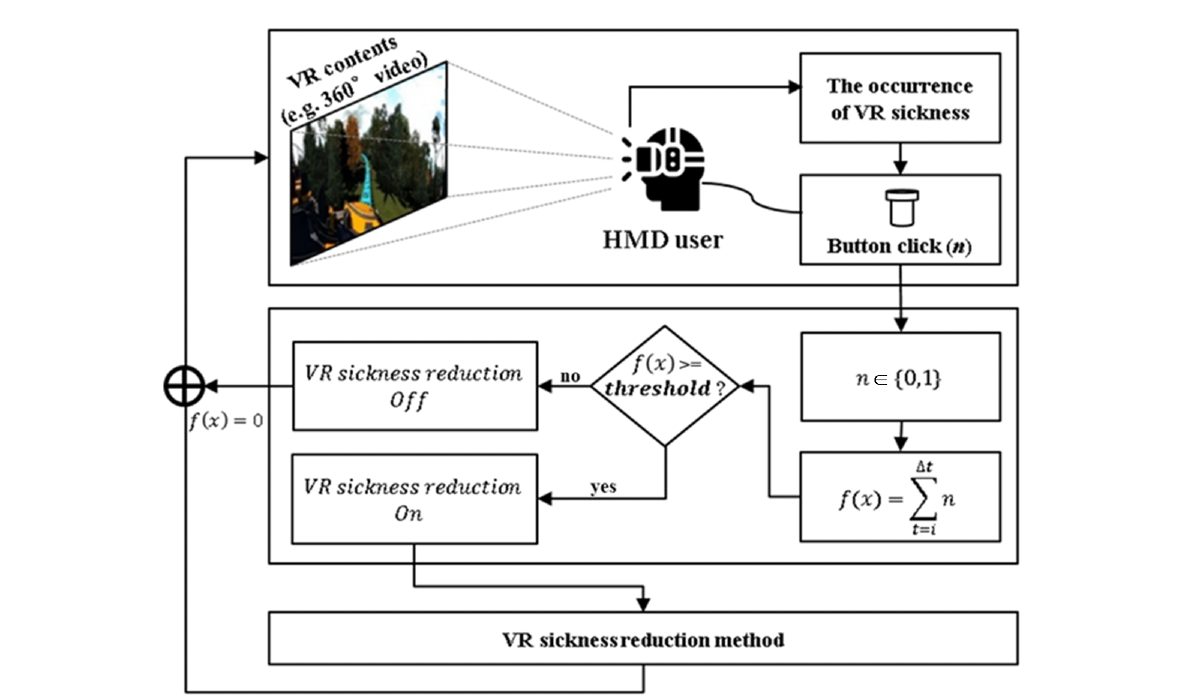
A conceptual diagram of a new approach proposed to reduce virtual reality (VR) sickness in real time. HMD: head-mounted display.

This section describes a new approach for real-time VR sickness reduction. In the proposed approach, when VR sickness occurs while the user watches VR content wearing an HMD, the user clicks a button to directly express the VR sickness responses. In this paper, the response directly expressed by the user is defined as the “VR sickness response.” Figure 1 shows a conceptual diagram of a new approach proposed to reduce VR sickness in real time. Users click a button when they experience VR sickness while wearing an HMD and watching VR content, and the sum of clicks becomes the VR sickness response. Then, the VR sickness reduction method is applied in accordance with the value of the VR sickness response. The following rules show how to apply a VR sickness reduction method in accordance with the VR sickness response:



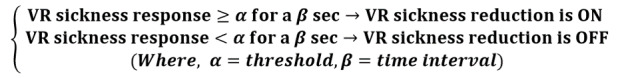



**Figure 2:**
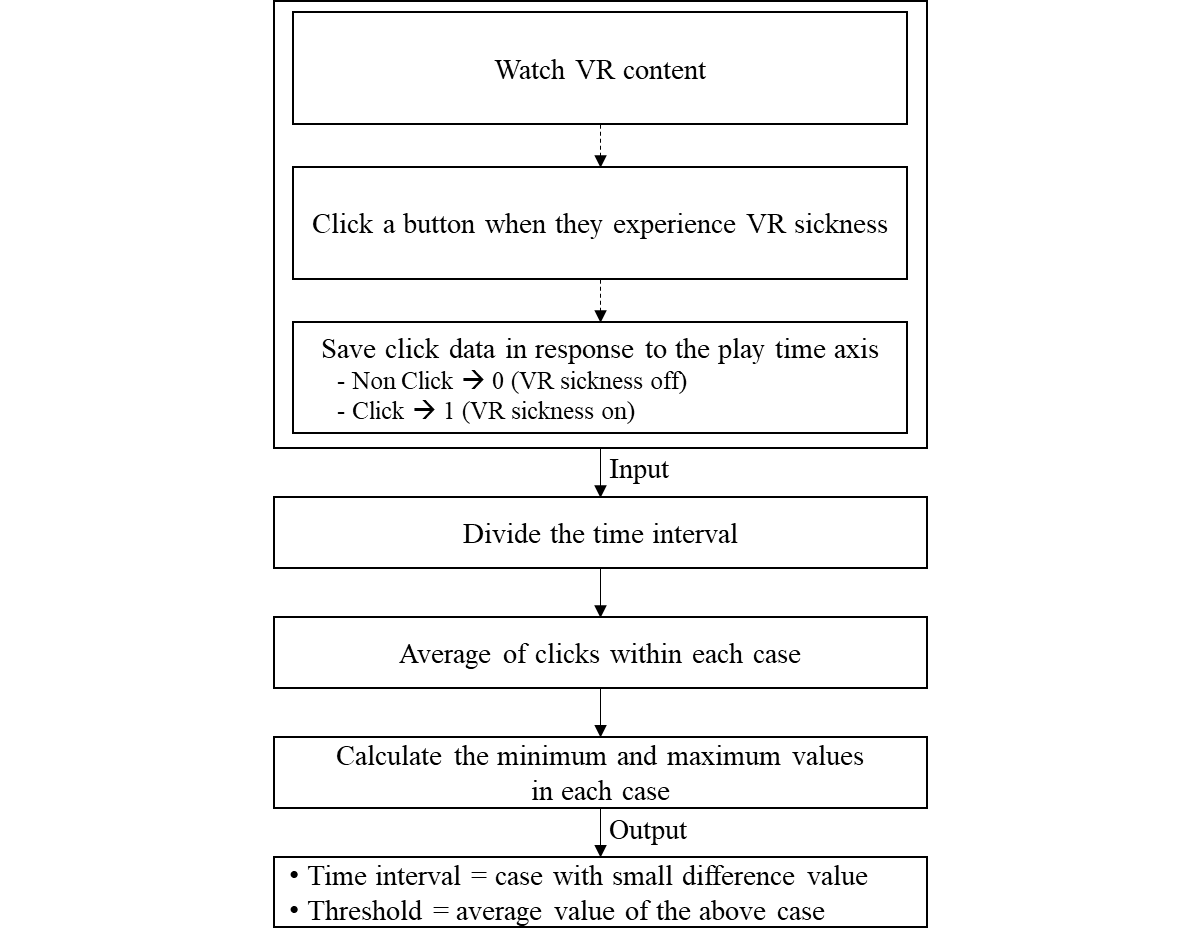
The Procedure for deriving the time interval and threshold. VR: virtual reality.

For example, if the VR sickness response is equal to or greater than the threshold (α in the equation) within a certain the time interval (β in the equation), the VR sickness reduction method is applied. Furthermore, if the VR sickness response is less than the threshold (α in the equation) during a certain the time interval (β in the equation) while the VR sickness reduction method is applied, the VR sickness reduction method is not applied. The parameters required to apply the proposed approach, the time interval, and threshold are derived from a preliminary experiment as shown in Figure 2.

The subject clicks a button when he/she experiences VR sickness while watching VR content. In addition, whether the subject clicked the button every 1 second is saved (clicked, 1; nonclicked, 0). For example, if video playtime is a total of 60 seconds, 60 of the data points expressed as 0 or 1 are saved. Using this data set, the time interval and threshold are determined by the following 4 steps.

First, the sections of VR content are evenly divided in accordance with the time interval of several cases (eg, the time interval=5, 10, 15…n). Second, the average of the subject’s VR sickness response in the evenly divided sections for each case is calculated. Third, the maximum and minimum values are selected from each case’s average, and the difference is calculated. Finally, the time interval of the case with the smallest difference between the maximum and minimum values is set as the time interval of the proposed approach, and the same case’s average VR sickness response is set as the threshold.

### Experiments

**Figure 3:**
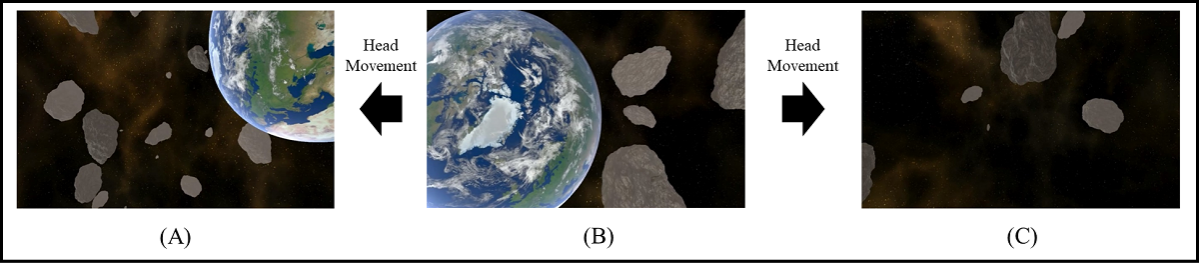
An example of the original virtual reality content: (A) and (C) are views changed in accordance with the user's head movement and (B) depicts the front view.

This section describes the experimental method for confirming the effectiveness of the proposed new approach to reducing VR sickness in real time. The experiment was conducted on 40 subjects (20 male and 20 female) in their 20s and 30s. In addition, as shown in Figure 3, a 3D VR space flight video was selected as the original VR content and it was named M0.

**Figure 4:**
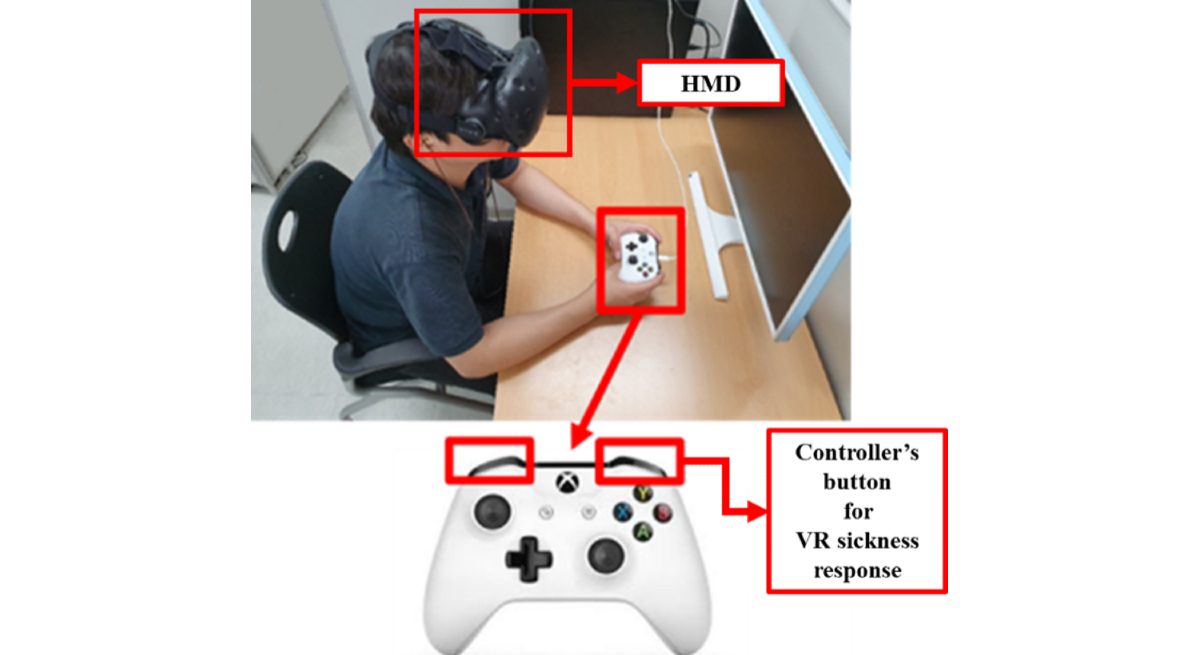
Experimental environment. HMD: head-mounted display; VR: virtual reality.

A view of original VR content is changed in accordance with the subject’s head movement (Figure 3B, which transitions to Figures 3A and 3C according to the head movement). Original VR content made subjects move the 3D space along a predetermined path for 60 seconds. In addition, acceleration, deceleration, and rotation (yaw, pitch, and roll) of the camera were applied to cause VR sickness. As shown in Figure 4, subjects watched VR content using HTC Vive Pro Eye, and they clicked the Xbox controller button to express their response to VR sickness in real time.

**Figure 5:**
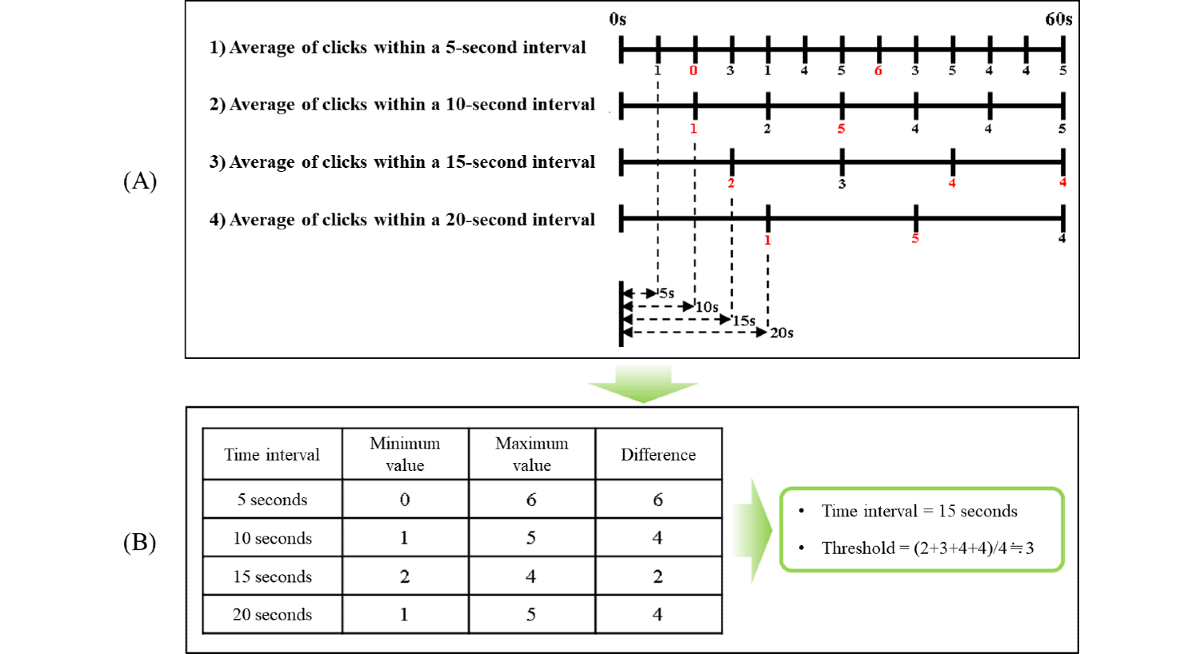
The time interval and threshold derived by the preliminary experiment: (A) are four cases of the preliminary experiment according to the time interval and (B) are the result of the preliminary experiment.

First, a preliminary experiment was conducted to derive the parameters of the proposed new approach (the time interval and threshold). In the preliminary experiment, VR sickness responses and SSQ for 40 subjects were measured. Figure 5 shows the time interval and threshold derived by the preliminary experiment. The original VR content used in the prior experiment were divided into a total of 4 cases in accordance with the time interval as shown in Figure 5A (the time interval of case 1 was 5 seconds; case 2, 10 seconds; case 3, 15 seconds; and case 3, 20 seconds). Then, in each case, the VR sickness response average value in the divided section was derived as shown in Figure 5B. As a result, the time interval of case 3, with the smallest difference between the maximum and minimum values of VR sickness response, was derived as the time interval of the proposed approach (time interval=15 seconds). In addition, the average value of VR sickness response in case 3 was derived as the threshold (threshold=3). After the parameters were derived, the VR sickness reduction method to be used in the proposed approach was selected. To this end, VR sickness reduction methods known to be effective were referred. Visual guide was a visual element that induces gaze movement and is known to be effective in reducing VR sickness [21]. In addition, it was known that the effect of VR sickness reduction is higher as the FOV decreases [22]. As a result, as shown in Table 1, a total of 3 VR sickness reduction methods were designed to be used in the proposed approach to reduce VR sickness in real time, and each method was named M1, M2, and M3.

The first VR sickness reduction method (M1) was applied with the visual guide, which had a 30% size of aspect ratio, and a position synchronized with the direction of the user’s head movement. The second VR sickness reduction method (M2) applied the FOV that had a size of 90° and a position synchronized with the direction of the user’s head movement.

When M2 is applied, if the subject moves their head from the center to the left of the screen, the FOV is limited on the basis of the left side of the screen.

The third VR sickness reduction method (M3) was applied, the FOV which had a size of 90° and a position synchronized to the direction of the user’s gaze movement. When M3 is applied, if the subject moves their gaze from the center to the left of the screen, the FOV is limited on the basis of the left side of the screen.

**Figure 6:**
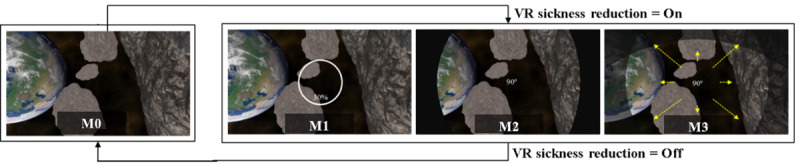
Examples of the virtual reality (VR) sickness reduction methods to be used in the proposed new approach (where, M0 is original VR content).

Figure 6 shows examples of the VR sickness reduction methods to be used in the proposed new approach.

**Figure 7:**
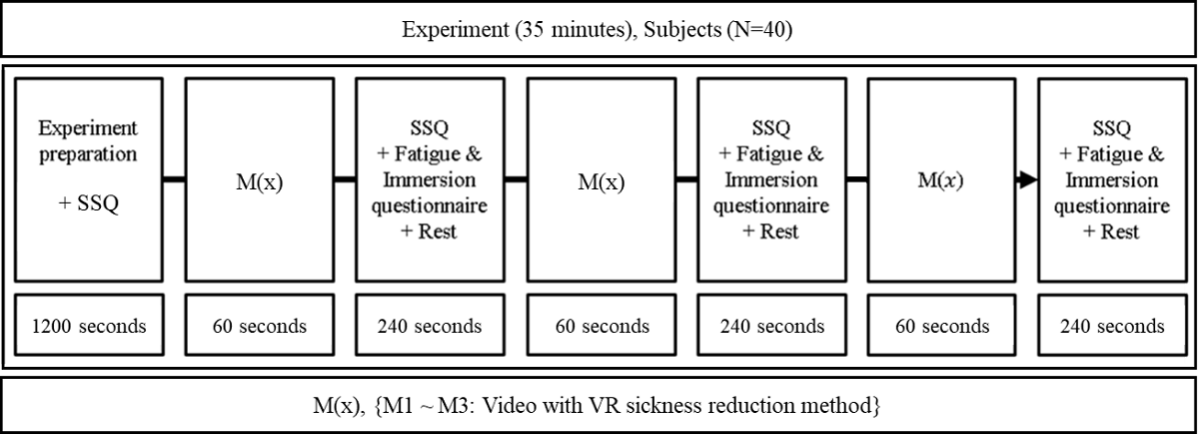
The experimental protocol. SSQ: simulator sickness questionnaire; VR: virtual reality.

The experiment was performed using the protocol shown in Figure 7 for 3 VR sickness methods (M1-M3). The subject responded to MSSQ and SSQ before the experiment, and after wearing the HMD, calibration was performed for eye tracking. The experiment was conducted for a total of 35 min, and each of the methods (M1-M3) was randomly followed to ensure reliability. The subject’s real-time VR sickness response was measured with respect to those 3 methods while watching VR content.

During the experiment, if the VR sickness response is measured more than 3 times within 15 seconds, the aforementioned 3 methods are applied.

Furthermore, the VR sickness reduction method is applied during the time interval, and if the VR sickness response is less than the threshold after the time interval, the method is discontinued.

After watching each VR content, subjects had time to respond to an SSQ and a questionnaire on fatigue and immersion; then, they had a period of rest to lower the level of VR sickness.

**Table 1:** Three virtual reality sickness reduction methods were used for the proposed approach.

Method	Feature	Property
M1	Visual guide 1	Size: 30% of aspect ratio Position: movement with the direction of head movement
M2	Field of view 1	Size: 90° Position: movement with the direction of head movement
M3	Field of view 2	Size: 90° Position: movement with eye tracking (every second)

### Ethics Approval

This study was approved by the institutional review board of Korea University of Technology and Education (approval 19090401).

## Results

This section describes the experimental results for confirming the effectiveness of the proposed new approach. In total, 26 out of 40 subjects did not experience VR sickness; hence, VR sickness reduction methods were not applied, and results based on 14 subjects who experienced VR sickness were analyzed. First, the SSQ scores of M0 and those of M1 to M3 were compared using a paired *t* test (2-tailed). As a result of the analysis, when VR sickness reduction methods (M1-M3) were used, the scores of nausea, oculomotor discomfort, and disorientation were significantly reduced (for all, *P*<.05).

**Figure 8:**
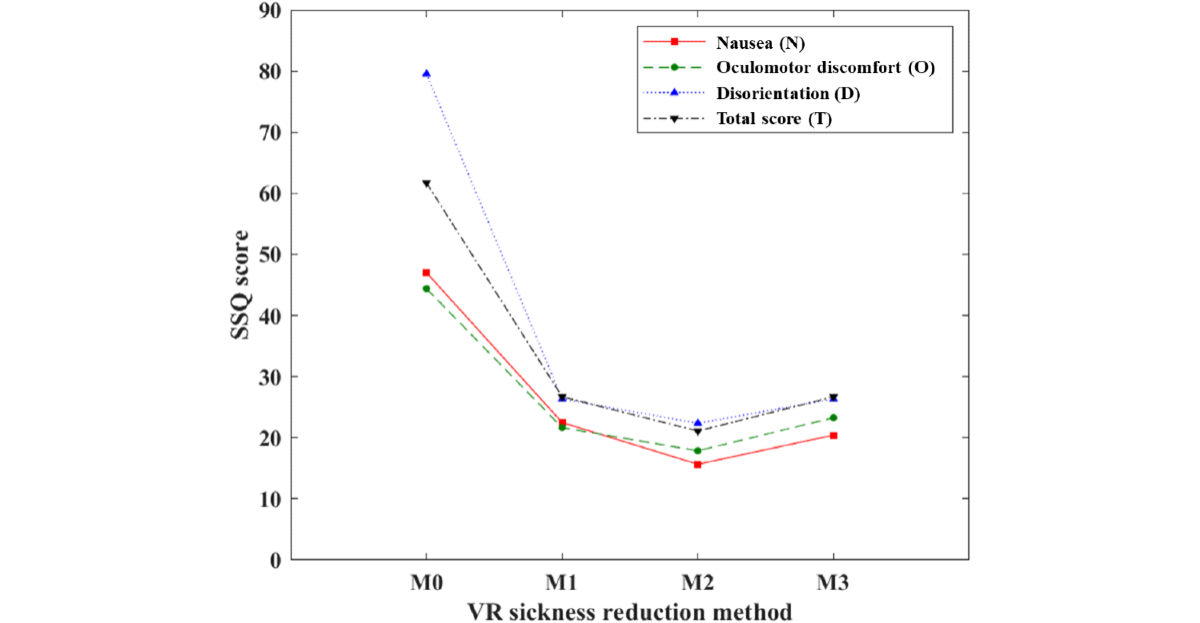
The simulator sickness questionnaire (SSQ) score of virtual reality (VR) sickness reduction methods (M).

The lowest SSQ score was observed in M2 and those of M1 and M3 were similar. Table 2 shows the results of VR sickness reduction in accordance with the SSQ score. Figure 8 shows the SSQ scores of the VR sickness reduction methods.

Furthermore, the VR sickness response of M0 and that of M1 to M3 were compared using a paired *t* test. All of the VR sickness reduction methods (M1-M3) showed significant VR sickness reduction.

Similar to the results of the SSQ scores, the lowest result was observed in M2, and the results of M1 and M3 were similar.

**Figure 9:**
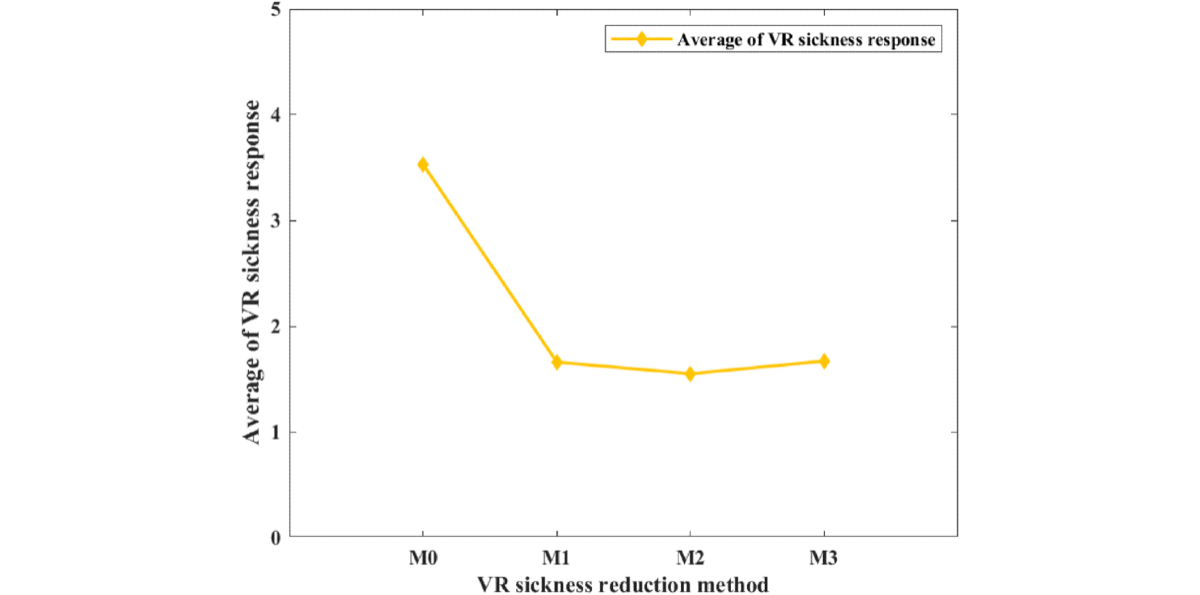
The average score of the virtual reality (VR) sickness response for the VR sickness reduction methods (M).

Table 3 shows the results of VR sickness reduction based on the VR sickness response. Figure 9 shows the VR sickness response to the methods.

**Figure 10:**
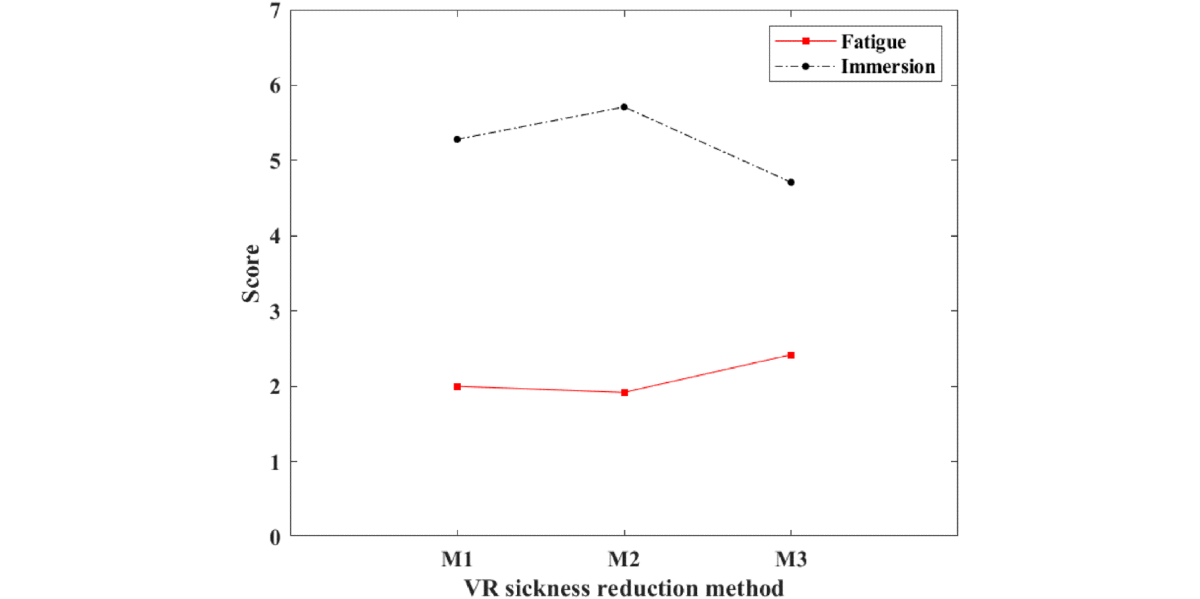
The questionnaire score for fatigue and immersion. VR: virtual reality. M: VR sickness reduction methods.

Finally, from the results of the fatigue and immersion questionnaire, it was confirmed that M3 caused the most fatigue and simultaneously caused the lowest level of immersion. Table 4 shows the questionnaire results for fatigue and immersion. Figure 10 shows the questionnaire score for fatigue and immersion.

**Table 2:** The results of virtual reality sickness reduction method based on the simulator sickness questionnaire score.

Method	Nausea				Oculomotor discomfort				Disorientation				Total score		
	Score	*t* test (*df*)	*P* value	Score		*t* test (*df*)	*P* value	Score		*t* test (*df*)	*P* value	Score		*t* test (*df*)	*P* value
M0	47.02	—^a^	N/A^b^	44.40		—	N/A	79.54		—	N/A	61.71		—	N/A
M1	22.49	2.917^c^ (13)	.01	21.66		3.261^d^ (13)	.006	26.85		3.241^d^ (13)	.006	26.71		3.230^d^ (13)	.007
M2	15.67	3.175^b^ (13)	.007	17.87		3.430^d^ (13)	.004	22.87		3.277^d^ (13)	.006	21.10		3.378^d^ (13)	.005
M3	20.44	2.385^c^ (13)	.03	23.28		2.668^c^ (13)	.02	26.85		3.061^d^ (13)	.009	26.71		2.766^c^ (13)	.02

^a^Not determined.

^b^N/A: not applicable.

^c^Significant at *P*<.05.

^d^Significant at *P*<.01.

**Table 3:** The results of virtual reality (VR) sickness reduction based on the VR sickness response.

Real-time VR sickness measurement	Score	*t* test (*df*)	*P* value
M0	3.53	—^a^	N/A^b^
M1	1.66	4.988 (13)	<.001
M2	1.55	3.706 (13)	.003
M3	1.67	4.645 (13)	<.001

^a^Not determined.

^b^N/A: not applicable.

**Table 4:** The questionnaire results for fatigue and immersion.

Methods	Fatigue	Immersion
M1	2.00	5.28
M2	1.92	5.71
M3	2.42	4.71

## Discussion

In this study, a new approach was proposed to reduce VR sickness in real time. The proposed approach used the VR sickness response, which is the result of the direct response of VR sickness that occurs while the user watches VR content wearing an HMD. Parameters necessary for the proposed approach were derived through preliminary experiments, and experiments were conducted to verify the effect of reducing VR sickness.

In addition, an experiment was conducted to confirm the effectiveness of the proposed approach, and from the experimental results, the following conclusions were drawn: (1) the methods (M1-M3) used previously [21,22] were effective in reducing VR sickness; (2) the VR sickness response can indicate the user’s VR sickness condition from the result that the subject’s VR sickness response and SSQ score have a similar pattern; (3) a new approach to providing a VR sickness reduction method based on the VR sickness response was significant; and (4) the VR sickness reduction method synchronized with gaze movement, which caused fatigue and reduced immersion.

From our results, it was confirmed that VR sickness could be measured without wearing equipment, and the VR sickness reduction method could be applied in real time in accordance with the user’s condition by the approach proposed in this paper. The proposed approach is expected to contribute to the spread of VR content by being applied to content streaming services.

This study has 2 limitations. First, a single type of VR content was used in the experiment; therefore, experiments using various types of VR content are needed to supplement the limitation. Second, the proposed method was applied after the user’s VR sickness occurred; hence, further studies attempting to predict and reduce VR sickness are required.

## Abbreviations

FOV: field of view
HMD: head-mounted display
MSSQ: Motion Sickness Susceptibility Questionnaire
SSQ: simulator sickness questionnaire
VR: virtual reality
